# Synthesis of High Molar Mass Poly(phenylene methylene) Catalyzed by Tungsten(II) Compounds

**DOI:** 10.3390/polym10080881

**Published:** 2018-08-07

**Authors:** Andreas Braendle, Carina Vidovič, Nadia C. Mösch-Zanetti, Markus Niederberger, Walter Caseri

**Affiliations:** 1 ETH Zürich, Department of Materials, Vladimir-Prelog-Weg 5, 8093 Zürich, Switzerland; andreas.braendle@mat.ethz.ch (A.B.); markus.niederberger@mat.ethz.ch (M.N.); 2 Institute of Chemistry, Inorganic Chemistry, University of Graz, Schubertstrasse 1, 8010 Graz, Austria; carina.vidovic@uni-graz.at (C.V.), nadia.moesch@uni-graz.at (N.C.M.-Z.)

**Keywords:** poly(phenylene methylene), tungsten(II) catalysts, high molar mass, fractionation, chain-growth polymerization, homoconjugation

## Abstract

Poly(phenylene methylene)s (PPMs) with high molar masses were isolated by polymerization of benzyl chloride catalyzed with tungsten(II) compounds and subsequent fractionation. Four different tungsten(II) catalysts were successfully exploited for the polymerization, for which a strict temperature profile was developed. The PPMs possessed roughly a trimodal molar mass distribution. Simple fractionation by phase separation of 2-butanone solutions allowed to effectively segregate the products primarily into PPM of low molar mass (*M_n_* = 1600 g mol^−1^) and high molar mass (*M_n_* = 167,900 g mol^−1^); the latter can be obtained in large quantities up to 50 g. The evolution of the trimodal distribution and the monomer conversion was monitored by gel permeation chromatography (GPC) and ^1^H NMR spectroscopy, respectively, over the course of the polymerization. The results revealed that polymerization proceeded via a chain-growth mechanism. This study illustrates a new approach to synthesize PPM with hitherto unknown high molar masses which opens the possibility to explore new applications, e.g., for temperature-resistant coatings, fluorescent coatings, barrier materials or optical materials.

## 1. Introduction

**Figure 1:**
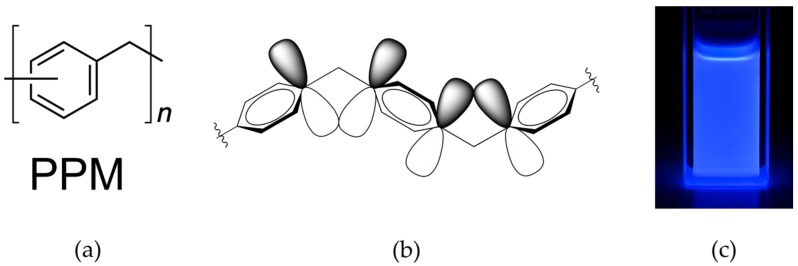
(**a**) chemical structure of poly(phenylene methylene) (PPM); (**b**) illustration of homoconjugation in PPM; (**c**) photograph taken under UV-light illumination (366 nm) of a polymer solution in chloroform in a 10 mm cuvette (PPM synthesized with the catalyst [W_2_Br_4_(CO)_7_], concentration of 83 mmol L^−1^).

Poly(phenylene methylene) (PPM) consists of phenylene rings bridged by a methylene unit (Figure 1a). PPM is completely amorphous [1,2,3,4,5] and possesses an exceptional combination of high thermal stability (onset of the decomposition temperature at 450 °C–470 °C) [1,2,3,6,7], and blue fluorescence in solution as well as in the solid state, with an unusually long photoluminescence lifetime (about 8 ns) and a high photoluminescence quantum efficiency compared to other polymers (41% in solid state) [7,8]. The fluorescence, however, is unexpected since in organic polymers this property is commonly a consequence of extended π–delocalization while the phenylene rings in PPM are electronically separated by methylene units. The results from thorough investigation of optical absorption spectra, photoluminescence spectra and photoluminescence excitation spectra at various conditions, supplemented by other observations, were in agreement only with homoconjugation as the origin of the photoluminescence (phenomena such as aggregation, π–π stacking or impurities could be excluded) [7]. Homoconjugation is a consequence of p-orbital overlap between isolated π-electron systems due to particular geometric situations, as schematically illustrated in Figure 1b for PPM. This type of conjugation is rare but established and has been identified so far mainly in low molar mass units [9,10,11,12,13,14,15,16,17], among others also in diphenylmethane [18,19], which can be considered as the simplest low molar mass analogue to PPM.

In this study, the focus is on the synthesis of PPM of higher molar masses. Namely, in spite of the fairly simple chemical structure of PPM and its promising properties mentioned above, PPM with number average molar masses (*M_n_*) above 12,500 g mol^−1^ or weight average molar mass (*M_w_*) above 52,500 g mol^−1^, respectively, have not been achieved so far (unless by fractionation which resulted in a product with *M_n_* of 61,200 g mol^−1^, however, in relatively low overall yield (20%) [1]).

The synthesis of PPM from benzyl alcohol and sulfuric acid was mentioned by Cannizzaro already in 1854 [20]. Subsequently, polymerizations were typically performed with benzyl chloride or benzyl alcohol as monomers and various Friedel Crafts catalysts (SnCl_4_, FeCl_3_, AlCl_3_) [1,5,21,22,23,24,25]. A selection of oxides (e.g., WO_x_, MgO) [26,27], zeolites [28,29], metals (e.g., Cu, Zn) [25,30], or complexes of transition metal carbonyls ([M(CO)_6_], M = Cr, Mo, W) [31,32] employed as catalysts for the polymerization of benzyl chloride resulted in PPM of rather low molar masses. In general, the reason for the low molar masses might originate from a step growth mechanism, which is known to generate high molar masses only with very pure monomers and monomer conversion above 99.0%; evidence for this mechanism was found at least for the PPM with the highest molar masses described so far [1].

In general, the poly(phenylene methylene)s hitherto obtained consist of a mixture of *ortho-*, *meta-* and *para*-substituted phenylene units. This is reflected in ^13^C NMR spectra, even though an unambiguous attribution of the individual substitution types to the signals has not yet been accomplished. The reports on the interpretation of the assignment of the signals in the region of the phenylene units are contradictory [33,34,35,36], yet in the region of the methylene units at least three groups of signals were identified [1]. These are related to pairs of substitution patterns which could, however, only partially be resolved (note that the chemical shift of a methylene unit is primarily influenced by the substitution patterns of the two phenylene units bound to the methylene unit). Remarkably, essentially the same ^13^C NMR spectra were found for a number of poly(phenylene methylene)s synthesized under different conditions and with different catalysts, and for products with different molar masses [33,34,35,36], suggesting that not only the substitution patterns but most likely also the polymerization mechanism was the same in those cases. Such polymers were reported to be strictly linear [4,36] and the dependence of the glass transition temperature on the molar mass follows the Fox–Flory equation [1,37], but nonetheless we would not exclude that some branching can exist in those polymers.

**Scheme 1:**
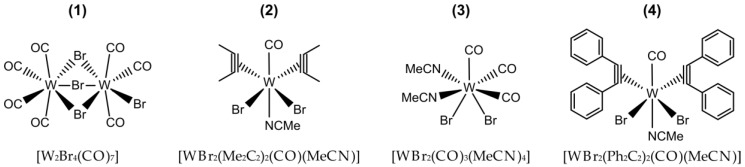
Chemical structure of the tungsten(II) complexes used as catalysts.

In this study, we pursue the synthesis of PPM catalyzed with tungsten(II) complexes. Compounds of this type can show good performance in other polymerization reactions such as ring opening metathesis polymerization (ROMP) [38,39]. Specifically, we employed four different organometallic tungsten(II) complexes shown in Scheme 1 [38,40,41,42]. Besides the molar masses, the type of basic reaction mechanism (step growth or chain growth) and the glass transition temperatures (and qualitatively the fluorescence) was also examined.

## 2. Materials and Methods

### 2.1. Materials

Chemicals were purchased from the following companies: Methanol, chloroform, 2-butanone from Sigma-Aldrich (Buchs, Switzerland); and benzyl chloride (99.5%) from Merck (Schaffhausen, Switzerland). With the exception of benzyl chloride (see below), all chemicals were used as received. All experiments for the preparation of the tungsten compounds were carried out under inert gas atmosphere employing standard Schlenk and glovebox techniques unless otherwise stated. Diphenylacetylene was recrystallized from ethanol prior to use. All solvents were purified by a Pure Solv Solvent Purification System.

### 2.2. Synthesis of the Tungsten Compounds

#### 2.2.1. [W_2_Br_4_(CO)_7_]

The catalyst [W_2_Br_4_(CO)_7_] was synthesized according to the literature [42].

#### 2.2.2. [WBr_2_(CO)_3_(MeCN)_2_]

This compound has been described previously [38] but was prepared by an alternative procedure: [W_2_Br_4_(CO)_7_] (17.565 g, 19.9 mmol, 1.0 eq.) was placed in an ice-cooled 250 mL Schlenk flask to which 200 mL of acetonitrile were slowly added. The suspension was allowed to warm up to room temperature and stirred for 1 h until a clear solution was obtained. After filtration over Celite, the solvent was reduced in vacuo to approx. 20 mL whereby deep red crystals appeared. They were isolated by filtration, washed with 10 mL acetonitrile and dried in vacuo to obtain the wine-red crystalline product (15.790 g, 30.9 mmol, 78%). Selected vibrations from infrared (IR) spectra (cm^−1^): ν 2319 (w, CN), 2291 (w, CN), 2024 (s, CO), 1908 (s, CO), 1896 (s, CO).

#### 2.2.3. [WBr_2_(Me_2_C_2_)_2_(CO)(MeCN)]

This compound has been described previously [40] by a slightly modified procedure: [W_2_Br_4_(CO)_7_] (2.100 g, 2.38 mmol, 1.0 eq.) was dissolved in 50 mL acetonitrile and filtered into a 100 mL Schlenk flask. The solvent was removed in vacuo and the residue dissolved in 45 mL CH_2_Cl_2_. The reaction mixture was cooled with an ice bath before 1.15 mL (14.7 mmol, 6.2 eq.) of 2-butyne were added dropwise. The flask was equipped with a bubbler and the mixture was stirred for additional 45 min and thereafter overnight at room temperature. After filtration, the solvent was removed in vacuo and the residue was recrystallized from 4 mL CH_2_Cl_2_ overlayered by 25 mL heptane to give 2.373 g (4.55 mmol, 96%) of the dark yellow crystalline product. ^1^H NMR *δ* (CD_2_Cl_2_): 2.83 (s, 6H, ≡–CH_3_), 2.85 (s, 3H, NC-CH_3_), 2.88 (s, 6H, ≡–CH_3_). ^13^C NMR *δ* (CD_2_Cl_2_): 5.04 (NC-CH_3_), 16.64 (≡–CH_3_), 19.75 (≡–CH_3_), 126.01 (CN), 157.73 (d, J_W-C_ =13.9 Hz, C≡C), 167.90 (d, J_W-C_ = 32.1 Hz, C≡C), 207.73 (d, J_W-C_ = 105.1 Hz, CO). IR (cm^−1^): ν 2034 (s, CO), 2298 (w, CN), 2326 (w, CN).

#### 2.2.4. [WBr_2_(Ph_2_C_2_)_2_(CO)(MeCN)]

This compound has been described previously [40] by a slightly modified procedure: [W_2_Br_4_(CO)_7_] (3.009 g, 3.41 mmol, 1.0 eq.) was dissolved in 60 mL acetonitrile and filtered into a 100 mL Schlenk flask. The solvent was removed in vacuo and the residue was dissolved in 60 mL CH_2_Cl_2_. The reaction mixture was cooled with an ice bath and 3.660 g (20.5 mmol, 6.0 eq.) of diphenylacetylene were added in portions. The mixture was stirred with an attached bubbler for additional 15 min and thereafter overnight at room temperature. After filtration, the volume was reduced to approx. 40 mL before 25 mL of heptane were added. Subsequent reduction of the volume to approx. 20 mL gave yellow needle-shaped crystals. After filtration and washing with heptane, 5.029 g (6.53 mmol, 96%) of product were obtained. ^1^H NMR *δ* (CD_2_Cl_2_): 2.27 (s, 3H, NC-CH_3_), 7.41 (m 20H, Ph). ^13^C NMR *δ* (CD_2_Cl_2_): 4.63 (NC-CH_3_), 126.84 (CN), 129.21 (2C, Ph), 128.98 (2C, Ph), 128.20 (2C, Ph), 128.11 (2C, Ph), 129.64 (Ph), 130.17 (Ph), 136.51 (Cq), 138.19 (Cq), 166.40 (d, J_W-C_ = 16.9 Hz, C≡C), 176.22 (d, J_W-C_ = 30.8 Hz, C≡C), 201.57 (d, J_W-C_ = 100.6 Hz, CO). IR (cm^−1^): ν 2301 (w, CN), 2328 (w, CN); 2075 (s, CO).

### 2.3. Synthesis of Poly(phenylene methylene) with Tungsten Catalysts

A general description is provided by the example of benzyl chloride polymerized with [W_2_Br_4_(CO)_7_]. First, benzyl chloride was exposed to vacuum (~10^−2^ mbar) for 8 h to remove the propylene oxide stabilizer (0.25% *w*/*v*). The removal of the stabilizer was verified by ^1^H NMR spectroscopy (disappearance of signals at 2.96 ppm, 2.73 ppm, 2.41 ppm, and 1.31 ppm). The catalyst [W_2_Br_4_(CO)_7_] (51.9 mg, 5.9·10^−2^ mmol) was placed under a nitrogen atmosphere in a 50-mL three-neck flask equipped with a mechanical glass stirrer. The destabilized benzyl chloride (12 mL, 104.8 mmol) was added at room temperature and the solution was stirred for 30 min. Subsequently, the solution was heated to 80 °C. Polymerization was carried out under a constant nitrogen flow rate of 17 mL min^−1^ to allow the evolved HCl to escape. After 24 h, the temperature was increased to 120 °C for another 24 h and subsequently to 160 °C for another 24 h due to increase in viscosity. The evolution of color during the reaction was as follows: clear yellow brown for the first minute, then dark blue, black at 80 °C, blue again at 120 °C, and eventually clear dark green at 160 °C. The resulting reaction mixture was allowed to cool down to room temperature and dissolved in 19 mL of chloroform, precipitated into 400 mL of methanol, filtered (cellulose filter), and dried under vacuum (10^−2^ mbar, 18 h) to give 7.3 g (82%) of green bluish PPM. ^1^H NMR (300 MHz, CDCl_3_, δ in ppm): 3.79 (br, 2H), 7.05 (br, 4H); elemental analysis: [C_7_H_6_]_n_ (molar mass: 90.12 g mol^−1^; calculated values in brackets, in % m/m): C 92.93% (93.29%), H 6.80% (6.71%). GPC (CHCl_3_): *M_n_* = 12,600 g mol^−1^, *M_w_* = 427,300 g mol^−1^, *M_w_*/*M_n_* = 33.9; DSC (T_g_): 52.0 °C.

The polymer syntheses catalyzed by the tungsten compounds [WBr_2_(CO)_3_(MeCN)_2_], [WBr_2_(Me_2_C_2_)_2_(CO)(MeCN)], and [WBr_2_(Ph_2_C_2_)_2_(CO)(MeCN)] were performed with the same procedure (and mass of catalyst) as described for the tungsten catalyst [W_2_Br_4_(CO)_7_]. The course of color during the reactions was similar for all syntheses, with the exception of the first minutes, where the color of the reaction mixture corresponded to the color of the added catalyst. The resulting molar masses are summarized in Table 1.

### 2.4. Synthesis of PPM for Reaction-Time-Dependent Analysis

The catalyst [W_2_Br_4_(CO)_7_] (336.5 mg, 0.380 mmol) was put under a nitrogen atmosphere in a 100-mL three-neck flask equipped with a mechanical glass stirrer. Benzyl chloride (78 mL, 681.2 mmol), after removal of the stabilizer as described above, was added at room temperature and the solution was stirred for 30 min. Subsequently, the solution was treated with the following temperature profile: 80 °C, 120 °C, 160 °C, and 200 °C; each temperature was hold for 24 h. Polymerization was carried out under a constant nitrogen flow rate of 17 mL min^−1^ to allow the evolved HCl to escape. During the reaction, 35 aliquots of ca. 1 g–1.5 g were removed from the reaction vessel (see Table S1 in the Supplementary Information for the exact time and amount of removal). A small part (ca. 100 mg) of these portions were dissolved in deuterated chloroform to immediately evaluate the state of monomer conversion and degree of polymerization (DP) by ^1^H NMR spectroscopy, as described previously [1]. The rest of the portions were then dissolved in chloroform (3 mL), precipitated into 20 mL of methanol, filtered through a cellulose filter and finally dried under vacuum (~10^−2^ mbar, 12 h). After four days, the remaining reaction mixture was allowed to cool down to room temperature and dissolved in 40 mL of chloroform, precipitated into 600 mL of methanol, filtered (cellulose filter), and dried under vacuum (10^−2^ mbar, 18 h) to give 21 g of green bluish PPM. GPC (CHCl_3_): *M_n_* = 10,800 g mol^−1^, *M_w_* = 202,400 g mol^−1^, *M_n_*/*M_w_* = 18.7.

### 2.5. Fractionation by Phase Separation

As an example, PPM (3.303 g) of *M_n_* = 12,600 g mol^−1^, *M_w_* = 427,300 g mol^−1^ and 2-butanone (70 mL) were mixed and stirred vigorously for 2 h, after which the suspension separated into a clear upper phase and turbid oily lower phase. The upper phase with the low molar mass polymer was decanted and concentrated with a rotary evaporator to approximately 5 mL. The solution was precipitated in 200 mL of methanol, and the solids were filtered and dried (as described above), to give 1.29 g (39%) of the polymer fraction **F1**. The fractionation procedure was repeated twice with the oily lower phase, using first 35 mL of 2-butanone for 1 h of stirring and then 35 mL of 2-butanone for 30 min of stirring. This yielded in 0.09 g (2.7%) of the polymer fraction **F2** and 0.02 g (0.6%) of the polymer fraction **F3**. These fractions (**F2** and **F3**) were not further investigated, since the yield was too low and the molar mass distributions too broad and therefore not of interest. The remaining oily lower phase was dissolved in chloroform and precipitated in 200 mL of methanol to give 1.70 g (51.3%) of the polymer fraction **F4**. The molar masses determined from GPC are as follows: **F1**: *M_n_* = 1600 g mol^−1^, *M_w_* = 4000 g mol^−1^ and **F4**: *M_n_* = 167,900 g mol^−1^, *M_w_* = 1,072,000 g mol^−1^.

### 2.6. Characterization

NMR spectra of the tungsten compounds were recorded using a Bruker Avance III spectrometer (Billerica, MA, USA). ^1^H NMR spectra were recorded at 300.13 MHz and ^13^C NMR spectra at 75.48 MHz. The chemical shifts δ are given in ppm. The multiplicity of peaks is denoted as broad singlet (bs), singlet (s), doublet (d), triplet (t), multiplet (m) and doublet of quadruplet (dq). Coupling constants J are given in Hertz. For ^1^H NMR and ^13^C NMR spectra of the polymers, a Bruker AV300 MHz instrument was used (solvent CDCl_3_). IR spectra were recorded in the solid state on a Bruker ALPHA-P Diamant ATR-FTIR (attenuated total reflection Fourier-transform infrared). Signal intensity was assigned as strong (s), medium (m) and weak (w). Elemental analysis was performed at the Microanalytic Laboratory of the Laboratory of Organic Chemistry (LOC) at ETH Zürich. For analysis by gel permeation chromatography (GPC) a Viscotek GPC system (Malvern, Worcs, UK) was used, equipped with a pump and a degasser (GPCmax VE2001; 1.0 mL min^−1^ flow rate), a detector module (Viscotek 302 TDA) and two columns (2× PLGel Mix-B; dimensions 7.5 mm × 300 mm) with chloroform as an eluent. Differential scanning calorimetry (DSC) was carried out with a Mettler Toledo DSC822^e^ instrument (Columbus, OH, USA), routinely calibrated against indium standards. Cooling and heating rates of 10 °C min^−1^ were applied, with all measurements performed under a nitrogen blanket.

## 3. Results

### 3.1. Polymerization

The conditions for the polymerization process of benzyl chloride catalyzed by the organometallic tungsten compounds (**1**)–(**4**) are based on a recent report about poly(phenylene methylene) with SnCl_4_ and FeCl_3_, respectively, as a catalyst, which provided the highest molar masses of isolated PPM so far [1]. Accordingly, the polymerizations were performed in a temperature range of 25 °C–200 °C without solvents and under a steady and controlled nitrogen flow to remove hydrogen chloride (HCl) which evolves during polymerization. Even at high temperature, the viscosity of the reaction mixture increased over the course of polymerization, hence mechanical stirring was required. Unlike in the addressed previous report, however, the reactions were carried out over a period of 4 d in which the temperature was gradually increased: 30 min at room temperature, 24 h at 80 °C, 24 h at 120 °C, 24 h at 160 °C, and 24 h at 200 °C. At the beginning of the reaction, an induction period was observed, in which the color of the reaction mixture changed from yellow brown to dark blue. During that phase, the polymerization of benzyl chloride did not start until the temperature was elevated to 80 °C (confirmed by ^1^H NMR spectroscopy of aliquots which were removed from the reaction mixture). The color change and the duration of the induction period clearly imply that the catalytically active species is not the originally added tungsten compound. Notably, to obtain high molar mass poly(phenylene methylene), it was crucial that the reaction mixture remained below 120 °C for 24 h. Otherwise, direct heating to 120 °C resulted in lower molar masses.

**Figure 2:**
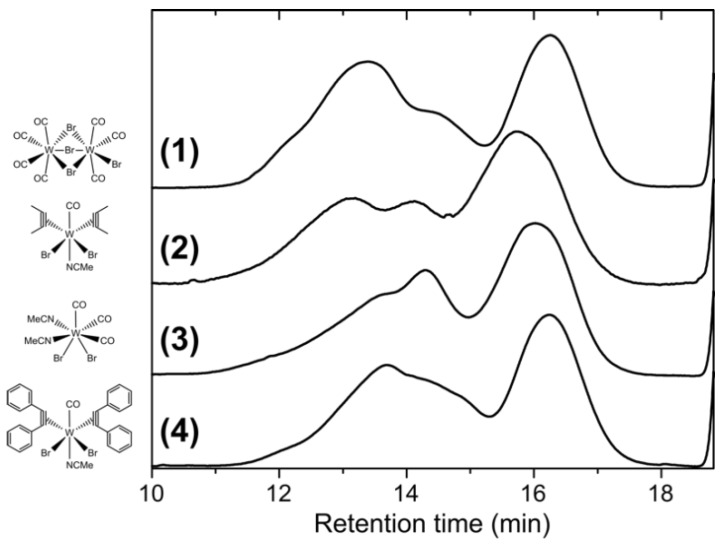
Comparison of the gel permeation chromatograms of PPMs synthesized with the catalysts (**1**)–(**4**), indicated with their chemical structures.

All polymers resulting from the four catalysts exhibit roughly a trimodal molar mass distribution with similar peak values, as shown in Figure 2. Accordingly, the molar mass distributions are very broad and the number average molar masses *M_n_* (8100 g mol^−1^–15,000 g mol^−1^) differ strikingly from the weight average molar masses *M_w_* (135,700 g mol^−1^–427,300 g mol^−1^) (see also Table 1). In particular, the *M_w_* are up to an order of magnitude above the values of the isolated PPM with hitherto highest molar masses (cf. Introduction). No difference in ^1^H and ^13^C NMR spectra was observed for the related polymers prepared with the four catalysts, i.e., those spectra were essentially superimposable. In addition, all polymers exhibited a pronounced fluorescence (an example is shown in Figure 1c); the fluorescence of PPM was already described extensively in the literature [1,3,7,8,21,36]. Furthermore, the UV/Vis absorption spectra of the polymers synthesized with the tungsten(II) catalysts showed the same characteristics as the products synthesized with SnCl_4_ or FeCl_3_ [7].

**Figure 3:**
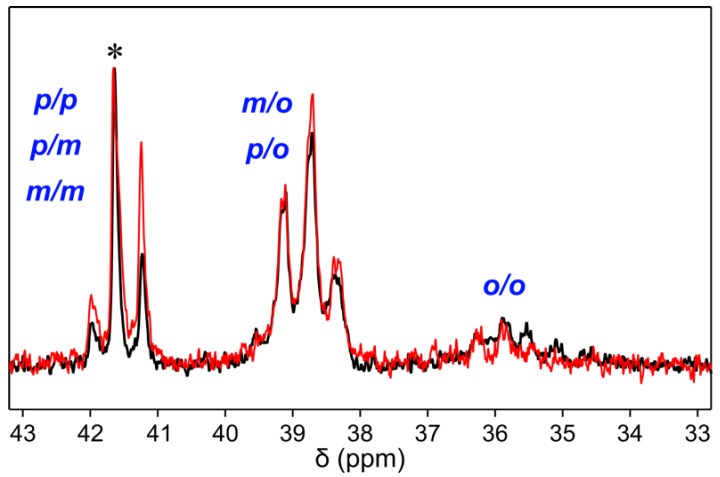
Comparison of the signals of the methylene unit in ^13^C NMR spectra for PPM synthesized with SnCl_4_ (black) and with [W_2_Br_4_(CO)_7_] (red). Based on the literature, the three peak groups are assigned to the respective substitution pattern of the two adjacent phenylene rings [1]. The spectra are normalized to the intensity of the peak marked with an asterisk.

Notably, when PPM synthesized with the tungsten catalyst [W_2_Br_4_(CO)_7_] was compared to a PPM synthesized with the Friedel–Crafts catalyst SnCl_4_ [1], the ^13^C NMR spectra revealed a small difference in intensity of the peaks representing the methylene units (Figure 3). The signal group specifically assigned to combination pairs of *para* and *meta* substitutions of the two adjacent phenylene rings (41 ppm–42 ppm) exhibited a decrease in relative intensities of two individual peaks, while the relative intensities within the other two signal groups (35 ppm–38 ppm and 38 ppm–40 ppm) did not change significantly. This suggests that the substitution patterns of PPM synthesized with tungsten(II) compounds are somewhat different from those of PPM synthesized with SnCl_4_ or FeCl_3_ as catalysts.

### 3.2. Fractionation

**Figure 4:**
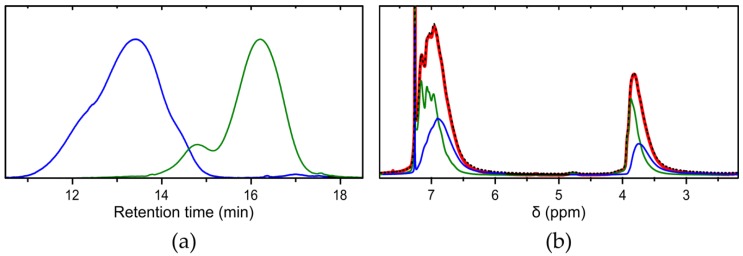
Separation of the trimodal distribution of the unfractionated PPM, synthesized with the catalyst [W_2_Br_4_(CO)_7_], eventually into a high and a low molar mass fraction, denoted as **F1** and **F4**, respectively. (**a**) gel permeation chromatograms of the low molar mass fraction (**F1**, green), and the high molar mass fraction (**F4**, blue); (**b**) ^1^H NMR spectra of the polymer **P** before fractionation (red) and the fractions **F1** and **F4**. Also included is the composed spectrum of the fractions according to their fractional yield (black dashed line).

It was shown recently that fractionation of PPM simply proceeds by dissolution of PPM in 2-butanone at high concentrations, which results in spontaneous separation in two phases containing PPM fractions of different molar masses [1]. Therefore, we attempted to isolate in particular the high molar masses of the trimodal molar mass distribution obtained with the tungsten(II) catalysts. As an example, the polymer obtained with the catalyst [W_2_Br_4_(CO)_7_] was selected for fractionation experiments. The pristine gel permeation chromatogram of the as-synthesized PPM, hereafter denoted as the unfractionated polymer **P**, is shown in Figure 2 (top line). The distinct trimodal distribution contains a high molar mass peak at retention time of 13.4 min with a clear shoulder at 14.5 min and a low molar mass peak at 16.2 min. In a highly concentrated solution of the unfractionated polymer (**P**) and 2-butanone, phase separation in a clear solution of low molar mass polymer (**F1**) and a swollen lump of higher molar mass polymer arose. This process of fractionation was repeated twice with the respective higher molar mass fraction to remove lower molar mass polymers (the intermediate fractions were not further investigated, since yields were too low and molar mass distributions broad, see Section 2 Materials and Methods). Eventually, the remaining polymer, which did not properly dissolve in 2-butanone anymore resulted in the high molar mass polymer **F4** (see also Section 2 Materials and Methods for more details). The fractional yields of the resulting polymers were 39.0% and 51.3% for the polymers **F1** and **F4**, respectively. The fractions **F1** and **F4** are displayed in Figure 4a as the low molar mass fraction (green line, *M_n_* = 1600 g mol^−1^, *M_w_* = 4000 g mol^−1^) and the high molar mass fraction (blue line, *M_n_* = 167,900 g mol^−1^, *M_w_* = 1,072,000 g mol^−1^), respectively. The corresponding peak positions of the unfractionated polymer closely matched those of the two fractions. The molar masses are summarized in Table 1.

The ^1^H NMR spectra of the two polymer fractions **F1** and **F4** exhibited a broadening of peaks from the low molar fraction **F1** to the high molar fraction **F4** (Figure 4b). This behavior is in accordance with their respective low or high molar masses, since in general signals in NMR spectra tend to broaden with increasing chain length of a polymer. In addition, a somewhat lower chemical shift was observed for high molar mass PPM because less repeat units are affected by the end group of the chain thus leading to a shift towards higher magnetic field strengths. Importantly, when the ^1^H NMR spectra of the polymer fractions are added in the proportions of their yields (0.415·**F1** + 0.550·**F4**), the sum of those spectra closely matches the spectrum of the polymer before fractionation (polymer **P**), as evident from Figure 4b (black dotted curve). This shows that the two fractions indeed represent the individual parts of unfractionated polymer **P**. Notably, since all polymer solutions were measured with the same concentration (14 mg of polymer and 1 mL of deuterated chloroform), all ^1^H NMR spectra in Figure 4b are displayed without normalization, bassline correction or phase corrections.

**Figure 5:**
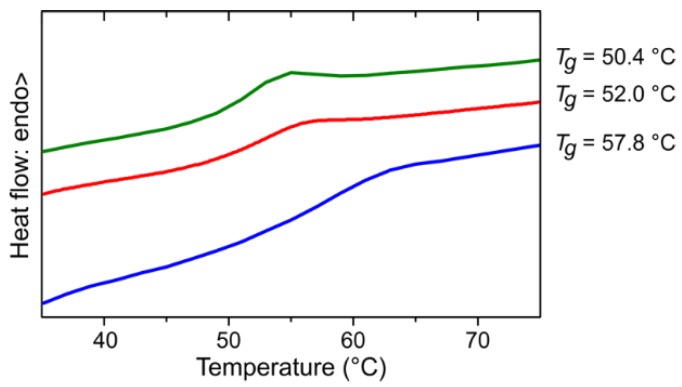
Differential scanning calorimetry thermograms (second-heating) for PPM synthesized with the catalyst [W_2_Br_4_(CO)_7_] (red) and the fractions **F1** (green) and **F4** (blue) thereof. The glass transition temperature (*T_g_*) is indicated at the right side of the respective curve. The color code corresponds to that of the colors of Figure 4.

The three polymers were also investigated with differential scanning calorimetry (DSC) (Figure 5). The glass transition temperature (*T_g_*) is clearly visible. The corresponding value of the unfractionated polymer **P** amounts to 52.0 °C, which is between the *T_g_* of the low molar mass fraction **F1** (50.4 °C) and of the high molar mass fraction **F4** (57.8 °C). An increase in *T_g_* with increasing molar mass is common and was also described for PPM synthesized with SnCl_4_ as a catalyst [1]. The *T_g_* of the PPM catalyzed with SnCl_4_ are slightly higher in spite of lower molar masses (ca. 66 °C for *M_n_* = 61,000 g mol^−1^ [1]), which could be due to differences in the substitution patterns of the polymers obtained under the action of the tungsten(II) catalysts and SnCl_4_, respectively (see above).

### 3.3. Evolution of the Course of the Reaction

**Figure 6:**
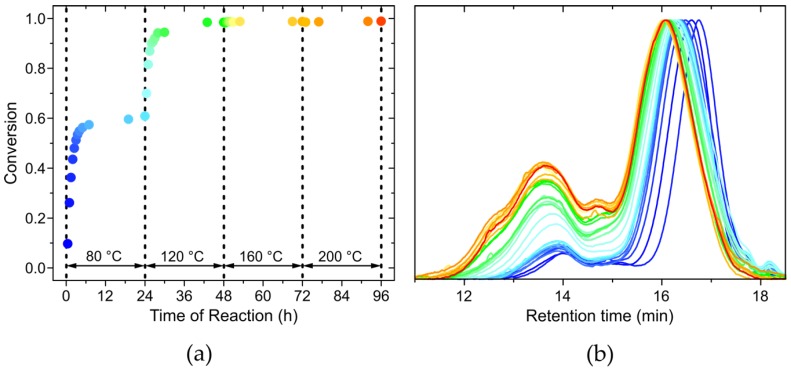
(**a**) progress of the monomer conversion (*p*) as a function of the reaction time. The temperature increments after 24 h from room temperature to 200 °C are indicated by the region separated by dashed lines; (**b**) gel permeation chromatograms of the aliquots taken during the reaction illustrate the evolution of the trimodal molar mass distribution over the course of the polymerization. The color code of the curves coincides with the colored dots in Figure 6a.

Since all the resulting polymers showed a broad molar mass distribution, and since their ^1^H and ^13^C NMR spectra did not differ considerably, the course of the reaction was followed with one example, namely the polymerization of benzyl chloride with the catalyst [W_2_Br_4_(CO)_7_], which exhibited the most pronounced separation between low and high molar mass peak in the GPC diagram. In order to monitor the monomer conversion (*p*) during polymerization and the evolution of the trimodal molar mass distribution during the course of the reaction, 35 aliquots were removed from the reaction mixture between 0.25 h and 96 h after the start of the reaction. A small amount of these aliquots was used to perform ^1^H NMR spectroscopy (see Figure S1 in the Supplementary Information), in order to determine the monomer conversion. The method to calculate the conversion was adopted from the literature (Equation (1)) [1], where both the chloromethyl peaks (R-CH_2_Cl) from the monomer and the end group appear at 4.6 ppm and the methylene peaks (R-CH_2_-R) from the polymer at 3.3–4.3 ppm. Figure 6a shows the progress of the conversion (rainbow colored dots) and the temperature (regions confined by dashed line) during the course of the reaction.

(1) $$p=\frac{\left[\left(R-{\mathrm{CH}}_{2}-R\right)\right]}{\left[\left(R-{\mathrm{CH}}_{2}-R\right)\right]+\left[\left(R-{\mathrm{CH}}_{2}\mathrm{Cl}\right)\right]}$$

Evidently, at 80 °C, a first plateau value was reached at a monomer conversion of 0.6 and the reaction did not proceed further until the reaction temperature was elevated. The polymerization suddenly resumed when the temperature increased to 120 °C and proceeded up to almost full monomer conversion. In agreement with this observation, the monomer peak disappeared in the ^1^H NMR spectra towards the end of the reaction (at temperatures of 160 °C and 200 °C). However, evaporation of some residual monomer cannot be excluded at 200 °C (the boiling temperature of benzyl chloride is 179 °C).

To analyze the evolution of the trimodal distribution of the molar masses, the above-mentioned aliquots were examined by GPC. For this purpose, the remaining amount of the aliquots was dissolved in chloroform and precipitated in methanol (as described in the Section 2 Materials and Methods). Figure 6b displays the chromatograms of the aliquots from 1 h after start of the reaction to the end of the reaction at 96 h. Already after 1 h, a peak emerged in the GPC diagram at a retention time of 16.8 min, representing polymer chains with molar masses of around 1200 g mol^−1^, and a smaller peak at 14.0 min representing polymers with molar masses of around 159,100 g mol^−1^. Over the course of the reaction, two basic changes were observed: the peaks shifted to higher molar masses (shorter retention times), and the high molar mass peak increased in intensity relative to the low molar mass peak. The low molar mass peak shifted from a retention time of 16.8 min (molar mass of 1200 g mol^−1^) to 16.1 min (molar mass of 4000 g mol^−1^), where the shift after the first reaction step (24 h at 80 °C) was only moderate. Hence, the low molar mass fraction was formed in particular during the first reaction step when monomer conversion was below 0.6. The high molar mass peak at a retention time of 14.0 min increased in relative intensity and shifted only little in the first stage of the reaction. In the second step of the reaction (temperature 120 °C), the relative intensity increased considerably. This trend continued up to the end of the reaction, although to a smaller extent. This increase of intensity went along with an increase of conversion from 0.6 to near completion (cf. Figure 6a).

Therefore, in general, lower temperatures obviously favor the formation of low molar mass polymers, where at temperatures above 120 °C higher molar mass polymers are preferred. However, as stated above, the reaction temperature had to remain at 80 °C for some time to achieve a large quantity of high molar mass polymers. The lower temperature in the early stage of the reaction might transform the tungsten catalyst precursor predominantly into an active catalyst, which catalyzes the polymerization to small molar masses. In a second step at temperatures above 120 °C, the already active catalyst or previously inactive species might be converted into other catalytically active tungsten compounds, which catalyze the reaction towards high molar mass polymers. In any case, we conclude that formation of catalytic species is a complex process, and probably more than one species is catalytically active. A certain evolution of tungsten species is also indicated by color changes during the reaction (yellow brown: room temperature, dark blue: 80 °C, dark green: 120 °C and higher). The multimodal molar mass distribution may reflect the difference in catalytically active species, but also different chain termination reactions of an individual catalytic species might contribute to the multimodal molar mass distribution.

The analysis of the course of the polymerization leads to the conclusion that the polymerization proceeds by a chain growth mechanism, in contrast to the step growth mechanism which was found for polymerization of benzyl chloride catalyzed by SnCl_4_ [1]. In the polymerization with the catalyst [W_2_Br_4_(CO)_7_], high molar mass polymers arose already at a monomer conversion of only 0.1 and a multimodal molar mass distribution was present already in an early stage of the reaction, both of which is in explicit disagreement with a step growth mechanism (represented by the Schulz–Flory distribution [43,44,45]). As an essentially trimodal molar mass distribution emerged upon the catalytic reactions with all tungsten complexes (**1**)–(**4**), it seems that chain growth mechanisms are involved in all cases. The reaction itself might proceed based on a Friedel–Crafts mechanism as proposed for polymerization of benzyl chloride with SnCl_4_ [5], however, with a (negatively charged) tungsten species interacting permanently with the end group of a growing polymer chain, in contrast to the case with SnCl_4_. Thus, polymerization with the tungsten compounds would be localized at a specific site during growth of a single chain, resulting in a chain growth mechanism.

## 4. Conclusions

The four tungsten(II) catalysts (**1**)–(**4**) are effective in polymerization of benzyl chloride to poly(phenylene methylene) (PPM), yielding isolated products far above the PPM of highest molar mass reported so far [1] and in higher yield (*M_n_* = 167,900 g mol^−1^, yield 51% vs. *M_n_* = 61,200 g mol^−1^, yield 20%). Notably, the polymerization can readily be performed in large quantities also on the laboratory scale. All four catalysts showed similar behavior in their catalytic activity. The resulting polymers showed a pronounced fluorescence and no significant difference in ^1^H NMR and ^13^C NMR spectra. The polymers directly obtained after synthesis exhibit roughly a trimodal molar mass distribution, which can be fractionated based on phase separation in concentrated solutions. Notably, the trimodal molar mass distribution of PPM obtained under the action of the tungsten(II) compounds (**1**)–(**4**) as catalysts and in particular the fact that high molar mass PPM forms already at a monomer conversion of 0.1, polymerization via chain growth mechanism is anticipated.

In contrast to PPM synthesized with catalysts like SnCl_4_ or FeCl_3_, a slight change in the substitution pattern (especially with regard to *meta* and *para* substitutions) was revealed with ^13^C NMR spectroscopy for the PPM synthesized with tungsten(II) catalysts. This difference in chemical structure might be the cause of the slightly lower glass transition temperature of 52 °C of high molar mass PPM synthesized with tungsten(II) catalysts compared to 66 °C of products synthesized with SnCl_4_.

With the isolation of poly(phenylene methylene) with molar masses of *M_n_* of 167,900 g mol^−1^ in high quantities, we believe in opening the application of this polymer for studies in various directions, ranging from fibers and coatings to optoelectronics.

## Figures and Tables

**Table 1:** Summary of number average molar mass (*M_n_*), weight average molar mass (*M_w_*) and polydispersity index (PDI) of the polymers synthesized with the four tungsten catalysts (**1**)–(**4**) (**top**) and of the two fractions **F1** and **F4** (**bottom**) from the fractionation of polymer **P**.

Polymer	*M_n_* [g mol^−1^]	*M_w_* [g mol^−1^]	PDI
[W_2_Br_4_(CO)_7_] (P)	12,600	427,300	33.9
[WBr_2_(Me_2_C_2_)_2_(CO)(MeCN)]	8100	135,700	16.7
[WBr_2_(CO)_3_(MeCN)_2_]	11,200	355,200	31.8
[WBr_2_(Ph_2_C_2_)_2_(CO)(MeCN)]	15,000	227,900	15.1
**Fractions of polymer (P)**			
**F1**	1600	4000	2.6
**F4**	167,900	1,072,000	6.4

## Supplementary Materials

The following are available online at http://www.mdpi.com/2073-4360/10/8/881/s1, Table S1: Temperature, amount, and conversion at the time of the removal of the aliquots; Figure S1: ^1^H NMR spectra of all aliquots resolved by time.
