# Supplementary material for: Synthesis of High Molar Mass Poly(phenylene methylene) Catalyzed by Tungsten(II) Compounds
Source: Polymers (Basel). 2018 Aug 7;10(8):881. doi: 10.3390/polym10080881 (PMC6404035; doi:10.3390/polym10080881)
Supplement: Supplementary file 1 [file polymers-10-00881-s001.pdf]

Supplementary Materials:

Synthesis of High Molar Mass Poly(phenylene methylene) Catalyzed by Tungsten(II) Compounds

*Andreas Braendle, Carina Vidovic, Nadia C. Mösch-Zanetti, Markus Niederberger, and Walter Caseri**

Table of Contents:

Table S1: Temperature, quantity, and conversion at the time of the removal of the aliquots.

Figure S1: ^{1}H NMR spectra of all aliquots taken during the course of the polymerization.

Table S1. Temperature, quantity, and conversion at the time of the removal of the aliquots from the reaction mixture catalyzed with $[W_2Br_4(CO)_7]$..

Aliquot #	Temperature [°C]	Amount [mg]	Conversion [%]	Time [h]
1	25	56.4	0.1	0.25
2	80	95.8	9.7	0.5
3	80	1204	26.1	1
4	80	1159	36.2	1.5
5	80	1569.8	43.5	2
6	80	1644.1	48.0	2.5
7	80	1632.3	51.2	3
8	80	1311.4	53.4	3.5
9	80	1206.7	54.8	4
10	80	1313.8	56.2	5
11	80	1665.7	57.4	7
12	80	1070.8	59.6	19
13	80	1128.1	60.8	24
14	120	1767.5	69.9	24.5
15	120	1564.5	81.5	25
16	120	1076	86.9	25.5
17	120	1038.1	90.0	26
18	120	1090.3	90.7	26.5
19	120	854.2	92.0	27
20	120	1243.6	94.1	28
21	120	1331.8	94.4	30
22	120	1116.4	98.4	43
23	120	844.1	98.4	48
24	160	803.3	98.2	48.5
25	160	807.1	98.5	49
26	160	803.3	98.7	49.5
27	160	893.3	98.7	50
28	160	882.1	98.6	51
29	160	1317.8	98.7	53
30	160	801.3	98.7	69
31	160	1872.4	98.7	72
32	200	948.4	98.5	73
33	200	925	98.6	77
34	200	911.2	98.8	92
35	200	1189	98.8	96

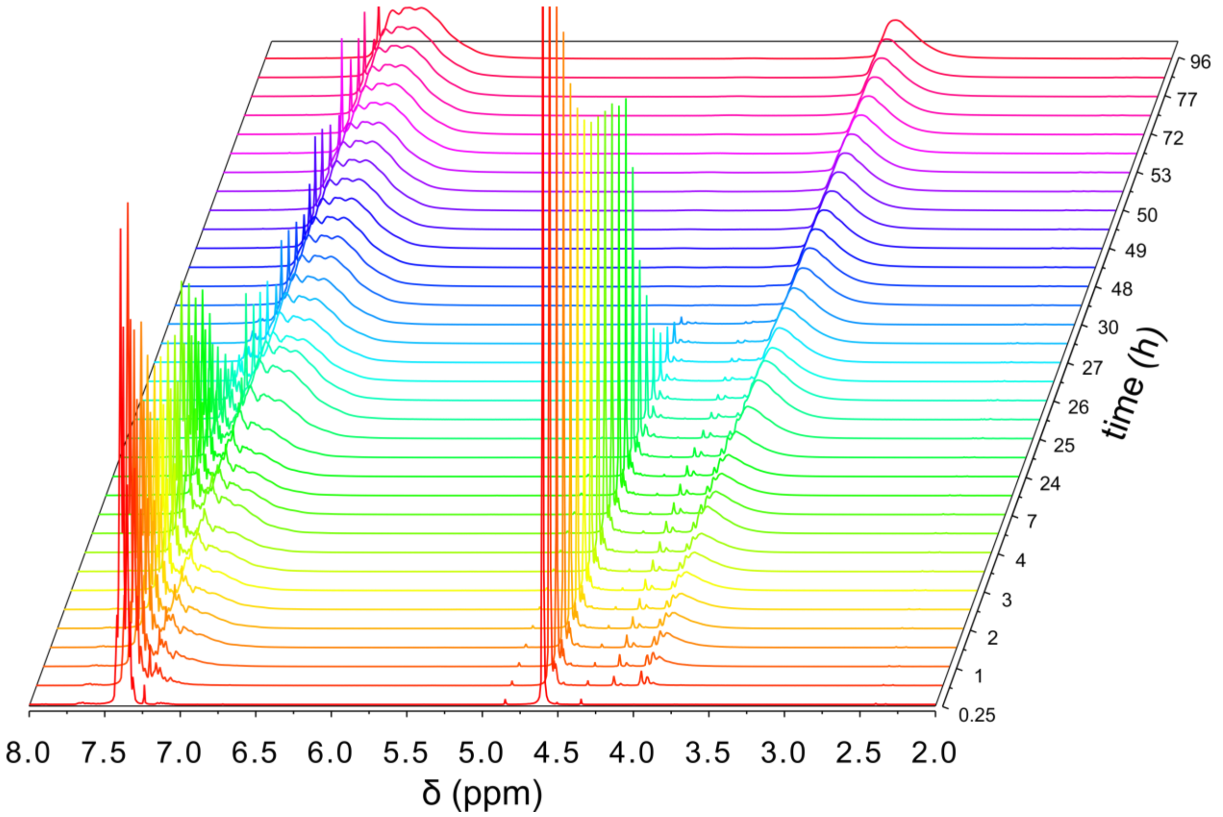

Figure S1. ^{1}H NMR spectra of all aliquots taken during the course of the polymerization of benzyl chloride with the catalyst $[W_2Br_4(CO)_7]$. These spectra were used to calculate the monomer conversion. The bottom spectrum was taken at the start of the reaction, the top spectrum at the end of the reaction after 96 h.
